# Prevalence of Soil-Transmitted Helminthiases and Schistosomiasis in Preschool Age Children in Mwea Division, Kirinyaga South District, Kirinyaga County, and Their Potential Effect on Physical Growth

**DOI:** 10.1155/2017/1013802

**Published:** 2017-08-23

**Authors:** Stephen Sifuna Wefwafwa Sakari, Amos K. Mbugua, Gerald M. Mkoji

**Affiliations:** ^1^Institute of Tropical Medicine and Infectious Diseases (ITROMID), Jomo Kenyatta University of Agriculture and Technology (JKUAT), P.O. Box 62000, Nairobi 00200, Kenya; ^2^College of Health Sciences, Jomo Kenyatta University of Agriculture and Technology (JKUAT), P.O. Box 62000, Nairobi 00200, Kenya; ^3^Centre for Biotechnology Research and Development, Kenya Medical Research Institute (KEMRI), P.O. Box 54840, Nairobi 00200, Kenya

## Abstract

Intestinal parasitic infections can significantly contribute to the burden of disease, may cause nutritional and energetic stress, and negatively impact the quality of life in low income countries of the world. This cross-sectional study done in Mwea irrigation scheme, in Kirinyaga, central Kenya, assessed the public health significance of soil-transmitted helminthiases (STH), schistosomiasis, and other intestinal parasitic infections, among 361 preschool age children (PSAC) through fecal examination, by measuring anthropometric indices, and through their parents/guardians, by obtaining sociodemographic information. Both intestinal helminth and protozoan infections were detected, and, among the soil-transmitted helminth parasites, there were* Ascaris lumbricoides* (prevalence, 3%),* Ancylostoma duodenale* (<1%), and* Trichuris trichiura* (<1%). Other intestinal helminths were* Hymenolepis nana* (prevalence, 3.6%) and* Enterobius vermicularis* (<1%).* Schistosoma mansoni* occurred at a prevalence of 5.5%. Interestingly, the protozoan,* Giardia lamblia* (prevalence, 14.7%), was the most common among the PSAC. Other protozoans were* Entamoeba coli* (3.9%) and* Entamoeba histolytica* (<1). Anthropometric indices showed evidence of malnutrition. Intestinal parasites were associated with hand washing behavior, family size, water purification, and home location. These findings suggest that* G. lamblia* infection and malnutrition may be significant causes of ill health among the PSAC in Mwea, and, therefore, an intervention plan is needed.

## 1. Introduction

Soil-transmitted helminthiases (STH) and schistosomiasis are listed among the many Neglected Tropical Diseases, with an established association to chronic, disabling, and disfiguring conditions occurring in settings of extreme poverty and even more so in rural poor and disadvantaged urban populations characterized by poor sanitation [1–3]. They contribute significantly to the burden of disease causing nutritional and energetic stress negatively impacting the quality of life and as such these parasitic infections have also been associated with malnutrition which contributes to more than one-third of all deaths of under-five children [2].

Estimates show that, in sub-Saharan Africa (SSA), about 198 million people are infected with hookworms [4], 192 million with schistosomiasis infection [5], 173 million with ascariasis infection [4], and 162 million with trichuriasis infection [4]. Based on the initial global percentage prevalence determined over 60 years ago [6] it is believed that the prevalence of STH has remained relatively constant in sub-Saharan Africa [4] where between one-quarter and one-third of sub-Saharan Africa's population is affected by one or more STH infections [4] with preschool age children and school age children carrying the highest prevalence and intensities [7, 8]. Available data estimates over 270 million preschool age children and over 600 million school age children live in areas characterized by intense transmission of intestinal parasites [9]. These infections have also been strongly associated with malnutrition [10] which is known to contribute to more than one-third of all deaths of under-five children [11].

In Kenya, the National Multi Year Strategic Plan for the Control of Neglected Tropical Diseases has prioritized intestinal worms among other NTDs (Neglected Tropical Diseases) as diseases of great public health importance mostly affecting the poorest of the poor [12].

Recent studies in Kenya estimate that about 6 million people are infected with schistosomiasis and even more are at risk [13]. The prevalence is set to range from 5% to over 65% in various communities in Kenya. It is endemic in 56 districts with the highest prevalence for* Schistosoma mansoni* occurring in lower Eastern and Lake Regions of Kenya and in irrigation schemes [14]. The Kenya Demographic and Health survey has also shown that 35.3% of under-five children were stunted nationwide, 6.7% were wasted, and 16.3% were underweight suggesting the significance of the burden of malnutrition particularly in rural Kenya [15]. To what extent the burden of malnutrition is contributed by intestinal parasites, in particular, helminth infections, remains to be accurately determined [16].

The prevalence of intestinal schistosomiasis, STH, and other intestinal parasitic infections in preschool age children (PSAC) in the Mwea rice irrigation scheme of Kirinyaga County in Central Kenya is not well documented, but, according to research done in an endemic community in Western Kenya, the prevalence in PSAC was demonstrated to be up to 37% [17] indicating the significant risk of infection in this age group in an endemic setup. Although there is a national school deworming programme which to date is still being implemented at the national level, the control programme has no clear policy for inclusion of PSAC (≤5 years old) in the mass treatment for STH and schistosomiasis. This thus highlights the need for a baseline survey to determine the prevalence, intensity, and possible effects on nutritional status of schistosomiasis and STH among other intestinal parasites in PSAC.

In view of the lack of information regarding the preschool age children, this study was undertaken to determine the prevalence of intestinal parasites in this age group, the risk factors favoring the spread of the parasites, and, subsequently, the possible association between the parasitic infections and the nutritional status.

## 2. Materials and Methods

### 2.1. Study Area

This study was conducted in the Mwea Division of Kirinyaga South district in Kirinyaga County, central Kenya (00°40′54′′S, 037°20′36′′E). This area is approximately 110 km North East of Nairobi and the main agricultural activity is rice farming which is grown under flood irrigation. Mwea is situated in the lower altitude zone (approx. 1150 mASL) of the district in an expansive flat land mainly characterized by black cotton and red volcanic soils. Mwea Division has a land area of approximately 542.8 sq. Km. Mwea Division has a population of 190,512 with an urban population of 7,625 (census, 2009). The specific area (survey area) of study was Thiba ward which comprises Nguka and Thiba sublocations of Kirinyaga County which is approximately 34 sq. Km with a population of 31,689. The nearest large town and administrative centre for Thiba ward is Wang'uru Town.

The geography of the area is mainly flat at an altitude ranging from 1150 to 1200 mASL

The area is mainly known for its horticultural crop farming where the main cash crop is rice grown under flood irrigation followed by maize.

The setting for the study site was largely a rural and peri-urban population.

### 2.2. Study Design

The study was a comparative cross-sectional study carried out to collect both quantitative and qualitative data. Based on the objectives, the study design investigated the possible association between infections and intensities of STH and schistosomiasis among other intestinal helminth infections on the one hand, with indicators of current physical growth status, on the other.

### 2.3. Study Population

The target population of the study was generally preschool age children between ≥2 and ≤5 years of age who have at least lived in the area under study for the past 6 months. Using a random sampling technique, the study selected 13 schools within the study area. The schools included Kandongu Primary School, Kiorugari Primary School, Mbui Njeru Primary School, Mukou Primary School, Ngurubani Primary School, AIPCA Primary School, Rurumi Nursery School, Thiba Primary School, Midland Day Care, Sibling Day Care, St Joseph Day Care, Thiba Glory Day Care, and Vision Day Care centres. Parents and guardians of all eligible children were invited to a meeting where, out of 517 parents in attendance, 361 consented to allow their children to participate in the study, and 361 children were enrolled into the study.

### 2.4. Data Collection

For every child recruited, a unique identifier number was assigned and information regarding the child/infant's name, sex and age, and area of residence (i.e., rural or urban) was collected. A questionnaire was also administered to consenting parents and guardian and was used to collect socioeconomic information of the parents/guardians and other behavioral information of the participating children considered to be relevant in contributing to the risk of infection.

#### 2.4.1. Questionnaire

Following the acquisition of an informed consent from the parents or guardians, questionnaires were administered to the parents of the enrolled children. The questionnaires were provided in both English and Swahili. The study also recruited translators in the local language (Kikuyu) to help parents better understand the questionnaire.

#### 2.4.2. Anthropometry

All children were examined by a qualified and registered community nurse/community health worker recruited by the study who carried out physical examination and measurements to obtain their weight, age, height, and mid-upper arm circumference. These parameters were collected as per the guidelines in the National Health and Nutrition Examination Survey's Anthropometry Procedures Manual developed by the United States Centre for Disease Control and Prevention (CDC). For purposes of accuracy, the instruments were calibrated regularly and random repeat measurements were done as a quality control measure. From the measurements, *Z*-score values for height-for-age (HAZ), weight-for-age (WAZ), and weight-for-height (WHZ) were calculated and used as indices for nutritional status.

#### 2.4.3. Stool Samples Collection and Examination

Each participant was provided with a stool sample collection container with unique identifiers, and with the help of activity coordinators approximately 4 grams (gm) of fresh stool sample was collected using polypots from each participating child.

From each sample collected, Kato-Katz thick smears were prepared for examination under a compound microscope. The fecal smears were prepared in duplicate on glass microscope slides to improve detection levels. The samples were processed within an hour of collection time. The Kato-Katz technique was mainly used to detect eggs and ova of* Schistosoma mansoni*,* Ancylostoma duodenale*,* Ascaris lumbricoides,* and* Trichuris trichiura*. Where infection was detected, intensity of infection was also noted and graded as either heavy, moderate, or low in accordance with the WHO proposed criteria [18, 19].

Further diagnosis using the formol concentration technique was done to detect presence of other intestinal parasites of public health significance that may have passed undetected in the Kato-Katz technique. Following diagnosis, subjects were divided into 3 groups: uninfected, infected with a single species, and infected with two or more species of intestinal helminthes.

### 2.5. Study Approval

The study protocol was approved by the Scientific and Ethics Review Unit of the Kenya Medical Research Institute. Approval to carry out the study in the area was also sought from administrative authorities in the schools, the Mwea Division Health Administration, and the Kirinyaga County Health Administration. Prior to enrollment of the study subjects, a meeting with parents/guardians of all eligible children was called with the help of the schools' administration, so that the study purpose, objectives, and procedures to be used could be explained including participants' rights if they both accept or decline to have their children participate in the study. Written informed consent was obtained and the children were recruited into the study. The parents/guardians were assured of the privacy and confidentiality of the information collected. All children found to be infected with intestinal parasitic infections received the appropriate medication prescribed by a qualified and registered clinician where albendazole (for soil-transmitted helminthes) and praziquantel (for schistosomiasis) were administered in their recommended doses as per the WHO recommendations [18]. Other infections or conditions were referred to the local health clinic.

### 2.6. Statistical Analysis

The data collected was first entered and stored into Microsoft Excel 2010. The data was verified and crosschecked for errors. A copy of the data was then recoded and exported into Statistical Package for Social Sciences (SPSS) Version 20 and baseline descriptive statistics were drawn.

Comparison of weight and height against infection status was done using independent *T*-test to assess significant differences in weight and height between the infected and the noninfected. ANOVA test was used to assess difference in height and weight between the noninfected, infected, and those with multiple infections.

Anthropometric data was exported to WHO Anthro [20] where WAZ, HAZ, and WHZ were derived and used to determine nutritional status. The anthropometric variables where applicable were reported as mean ± standard deviation (SD) 95% confidence interval.

Based on the *Z*-score values obtained for WAZ, HAZ, and WHZ, the children were categorized as normal (≤2 and ≥−2 *Z*-score), underweight (≥−3 and <−2 *Z*-score), or severely underweight (<−3 *Z*-score); stunted (≥−3 and <−2 *Z*-score) or severely stunted (<−3 *Z*-score); and wasted (≥−3 and <−2 *Z*-score) or severely wasted (<−3 *Z*-score).

Binary variables were compared using Student's *t*- test and Chi-square test where applicable.

Demographic and socioeconomic data were entered as categorical variables and the frequencies and percentages were calculated. Later they were assessed using a binary logistic regression model with the baseline category as the least likely to result to an infection outcome.

All statistical tests were evaluated for significance at *P* < 0.05; 95% CI (confidence interval).

## 3. Results

### 3.1. General Characteristics of the Study Group

A total of 361 children were recruited into the study of which 50.40% were male (*n* = 183) and 49.60% female (*n* = 178). The mean age in months was 46.62 ± 9.68 (45.62–47.62), 95% CI. Mean height was 101.78 ± 6.57 cm (101.10–102.45), 95% CI, and mean weight was 14.71 ± 2.08 kg (14.49–14.92), 95% CI.  Table 1 gives an overall summary of the study group demographics while Table 2 provides an age group sex distribution of the population.

The same number of families participated in the questionnaires determining behavioral trends and socioeconomic status and summary of the responses is tabulated on Table 3.

### 3.2. Parasitological Investigations

Out of the total 361 children enrolled in the study, 108 children (29.9%) were found to be infected with an intestinal parasite of which 15 (3.9%) had multiple parasite infections. Prevalence of each parasitic infection is shown in Table 4. The prevalence of* Ancylostoma duodenale* was at 0.6%,* Ascaris lumbricoides* 3.3%,* Entamoeba histolytica* 0.3%* Enterobius vermicularis* 0.83%,* Entamoeba coli *3.88%,* Giardia lamblia* 14.68%,* Hymenolepis nana* 3.6%,* Schistosoma mansoni* 5.54%, and* Trichuris trichiura* 1.11%, combining single and multiple infections. It was noted that prevalence for most infections showed a tendency to increase with age as is illustrated in Table 5. There was a significant difference in prevalence of* Schistosoma mansoni* infection between boys and girls where boys showed a higher tendency to be infected with schistosomiasis (*t* = 3.308; *P* = 0.03; 0.026–0.119 at 95% CI). All other infections showed no statistically significant difference between boys and girls. Generally, infection prevalence showed tendency to increase with age. Based on independent *t*-tests done to compare weights and heights of those infected versus the uninfected, there was no statistically significant difference based on the overall infection status (weight: *P* = 0.07482, *t* = 1.6520; height: *P* = 0.2230, *t* = 1.6519); there was however statistical significant difference in weight between those infected with* Giardia lamblia* and those not infected (*P* = 0.0362, *t* = 1.8015). All other infections individually showed no significant difference in weight and height between those infected and the noninfected.

### 3.3. Nutritional Status

#### 3.3.1. Weight and Height

Based on the weight for height of the children, the prevalence of malnutrition was determined and is presented in Table 6. The mean weights of the participants (*n* = 361) were 14.71 kg (14.49–14.92), 95% CI, and height was 101.78 (101.10–102.45) 95% CI. The mean heights and weights of the children showed no statistical difference between males and females.

Prevalence of severe stunting, severe underweight, and severe wasting were 0.6% (2) (−0.2–1.3—95% CI), 1.7% (6) (0.3–3.0—95% CI), and 3.6% (13) (2.1–6.2—95% CI), respectively

Seven boys and 8 girls were found to be severely wasted, 1 boy and 1 girl were severely stunted, and 4 girls and 2 boys were severely underweight. The prevalence of wasting, underweight and stunting was also noted to increase with age. There was also significant difference in HAZ (*P* = 0.036, *t* = 2.108, 95% CI = −0.6486–−0.2251) and WHZ (*P* = 0.022, *t* = 2.303, 95% CI = 0.0372–0.4738) between boys and girls. The results of height and weight and prevalence of malnutrition are shown in Tables 6 and 7.

Based on the general status of infection of the children, there was a significant difference in WAZ (*P* = 0.000;  *t* = 3.675; 95% CI = 0.2162–0.7175) and HAZ (*P* = 0.001; *t* = 3.383; 95% CI = 0.2438–0.9210) between the infected and the noninfected for all parasitic infections. With regard to specific infections, children with* Giardia lamblia* infections showed significantly lower mean weights (14.14 versus 14.80 kg; *P* = 0.031, *t* = 2.171; 95% CI = 0.0626–1.2669), mean weight for age *Z*-scores (−1.275 versus −0.542; *P* = 0.000, *t* = 4.728; 95% CI = 0.4285–1.0387), and mean height for age *Z*-scores (−0.7582 versus 0.2776; *P* = 0.000, *t* = 4.728; 95% CI = 0.6075–1.464) when compared to the noninfected children.

**Figure 1:**
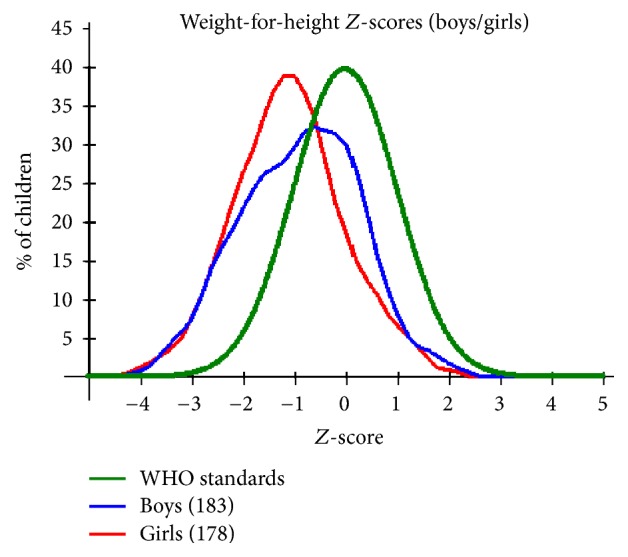
A plot of weight for height *Z*-scores by gender for the PSAC in Mwea Division against the recommended WHO standards.

Based on the sex of the children with regard to wasting, both boys were affected with boys showing slightly higher degree of severe wasting in contrast to girls who show slightly higher number of moderate wasting. Comparison by a Student's* t*-test showed that the slight difference was of no statistical significance. Table 6 gives a summary of the percentages of children affected with malnutrition. In Figure 1 it further shows that the majority of girls although within normal limits, that is, *Z*-score values within the normal limits of 2 standard deviations, showed a tendency to deviate towards the negative with a mean *Z*-score value of −1.10. This is likely as a result of the many of the girls recording lower weight to height *Z*-score values although within the normal interval. On the other hand, majority of boys within the normal WHO confidence interval recorded *Z*-score values closer to the WHO mean *Z*-score value. Figure 1 also draws attention to the percentage of children falling outside the −2 Standard deviation mark indicating percentage of children with wasting.

**Figure 2:**
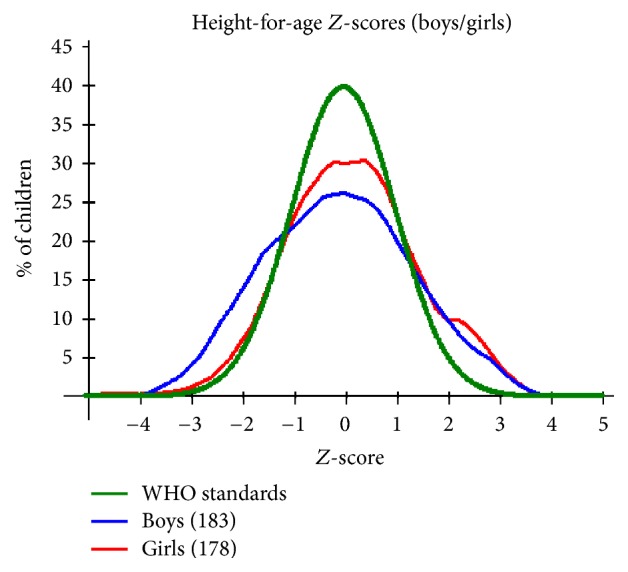
A plot of height for age *Z*-scores by gender for the PSAC in Mwea Division against the WHO recommended standards.

With regard to height for age, Figure 2 is indicative of more boys affected by stunting with 8.2% of the boys being moderately stunted compared to 3.4% of girls, that is, percentage of children falling outside the −2SD WHO standard interval. As for severe stunting, boys again showed slightly higher percentage compared to girls. This was confirmed by the Student* t*-test which showed a statistically significant difference in HAZ (*P* = 0.036, *t* = 2.108, 95% CI = −0.6486–−0.2251) between the boys and the girls.

**Figure 3:**
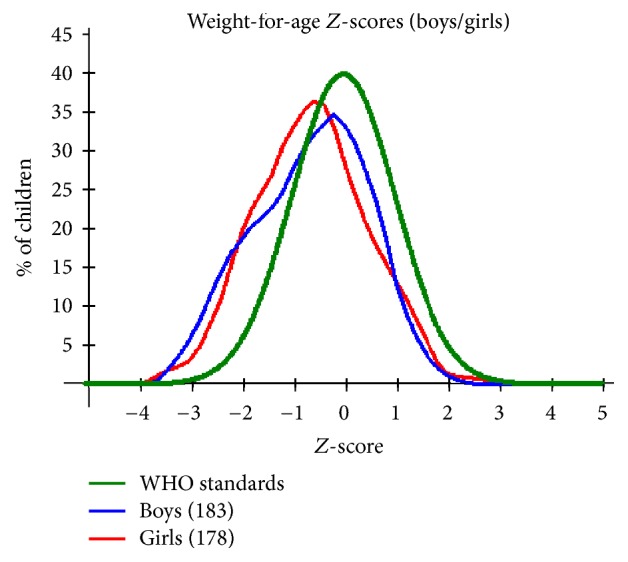
A plot of weight for age *Z*-scores by gender for the PSAC in Mwea Division against the WHO recommended standards.

The weight for age *Z*-score values show boys to be slightly more affected by malnutrition with a percentage of 14.2% compared to girls, 11.8%. The same trend is observed with severe malnutrition as is shown in Table 6. As per Figure 3, the boys' curve shows some degree of skewness to the left although it is centered towards the mean and the skewness translates to the slightly higher percentage of boys affected by malnutrition. This is confirmed by Student's* t*-test showing statistically significant difference in WHZ (*P* = 0.022, *t* = 2.303, and 95% CI = 0.0372–0.4738) between boys and girls. As for the girls' curve, there is tendency to slightly shift towards the left which is indicative of the girls being centred towards the negative side of the WHO mean.

With regard to socioeconomic and demographic factors, the mean weight of the children was found to be significantly lower among those whose parents had other children above the age of 5 years (weight: 15.021 kg Vs 13.96 Kg; 95% CI = 0.5931–1.51168; *t* = 4.507; *P* = 0.000). A look at the summary of the socioeconomic and behavioral characteristics of the study population (see Table 3) focusing on factors that may have an influence on the infection and nutritional status of the target study group showed that 68.4 percent of the sampled population proved to be aware of the ways to prevent transmission of intestinal parasites. However, a vast majority fall short of applying preventive measures most of who lack the means to implement such measures.

A binary logistic regression model performed to ascertain the effects of demographic, behavioral, and socioeconomic status of the population on the children's infection status was statistically significant, *χ*^2^ = 104.4, *P* = 0.000. It explained 35.6% (Nagelkerke *R*^2^) of the variance in infection and correctly classified 78.1% of the cases. The model revealed that the infection status of children was significantly influenced by their hand washing behavior, their water purification method, classification of home location, and whether the family had other children above the age of 5. Children who reported never washing hands on the key recommended times were 6.4 times likely to be infected (odds ratio (OR) 6.4, *P* = 0.010, 95% CI), children in families with siblings above 5 years were 2.6 times more likely to be infected with a parasitic infection (OR: 2.565, *P* = 0.07, 95% CI), and those families that reported not using any water purification methods were 3.6 times more likely to be infected (OR 3.602, *P* = 0.08, and 95% CI) while children living in the rural areas were at a 8.1 times (OR 8.051, *P* < 0.001, and 95% CI) higher risk of infection with a parasitic infection.

## 4. Discussion

Parasitic infections are well known for their burden of disease mainly attributed to their chronic and insidious impact on the health, nutrition, and quality of life of those infected rather than to the mortality they cause [21]. The study showed that 29.9% of the children were infected with various parasitic infections. The prevalence of specific parasitic infections was generally low with prevalence of below 6%. However, a prevalence of 15% for* Giardia lamblia, *a parasite often associated with diarrhea and acquired through drinking contaminated water and consumption of contaminated soil or food [22], was interesting but not surprising. This finding suggests that this parasite is most likely common in this area and a cause of ill health among children of 5 years of age or less, in this area. Since there were no previous studies to investigate their prevalence, this study served as a baseline survey providing information on the status of infection in PSAC. The study was also able to demonstrate that 3.6%, 1.7%, and 0.6% of the children were severely wasted, underweight, and stunted. Based on the general infection status, there was a significant difference in WAZ (*P* = 0.000;  *t* = 3.675; 95% CI = 0.2162–0.7175) and HAZ (*P* = 0.001; *t* = 3.383; 95% CI = 0.2438–0.9210) between the infected and the noninfected. The study demonstrated a significant lower mean weights, mean weight for age, and mean height for age for children infected with* Giardia lamblia* infection, a clear indication of the impact of* Giardia lamblia* on the nutritional status of children [22]. Other studies have also documented similar findings with regard to the effects of* Giardia lamblia* on weight and height of children [22] where chronic infections with giardia lamblia have been associated with clinical manifestation of malnutrition. The study however could not demonstrate statistically significant association linking other specific parasitic infections to malnutrition. This could be attributed to the low prevalence of these infections. This study has also shown that hand washing behavior, water source for drinking, water purification methods, and classification of home location and family size were strongly associated with the general status of infection. Similar studies have also demonstrated association between soil-transmitted helminth infection with water supply source, hand washing behavior, and family size [23].

The results of the binary logistic regression in Table 7 show that the transmission of* Schistosoma *spp., STH, among other parasitic infections have been strongly associated with sanitation and hygiene and the lack of clean and safe water supply. Most of these conditions have mostly been linked to poverty as the root cause and as such have been linked to malnutrition and many other health problems including parasitic infections [2, 16]. Of the total number of infections 93.5% (101 children) occurred in the rural setting and only 6.5% (7 children) occurring in the urban setting. Also from the regression analysis the odds of a child living in rural areas is up to 8.1 times higher (See Table 7) compared to the children in urban settlement. This presents a clear association of infection with the rural setting which is well known to be associated with poverty and lack of access to clean and safe water [23, 24].

The study findings of the study have also demonstrated an association between malnutrition and family size where families with more than 3 children above the age of 5 had a lower mean weight compared to families with <3 children. Other studies have demonstrated this to be especially common in rural and poor socioeconomic communities due to inadequate distribution of food among family members [2]. Also to note is the association between families where children have siblings above the age of 5 had a higher risk of infection which presents a likelihood of infection being transmitted from older siblings to younger ones.

Regardless of infection status, the study populations showed high prevalence of malnutrition, with prevalence and severity showing tendency to increase with age as is illustrated in Table 8. This observation is consistent with findings from other studies [2] that demonstrated significant increase of risk of malnutrition with increase in age for children under 5. These observations could as well be attributed to poverty and other health problems which do not exclude other parasitic infections beyond the scope of this study. Figures 1, 2, and 3 provide a graphical representation of the nutritional status of the preschool age children in Mwea Division.

The deviation observed for WHZ scores showing skewness to the left (negatively skewed) and a shift to the left (see Figure 1) is indicative that many of the children deviate negatively from the WHO standard WHZ means. Low weight for height *Z*-scores is known to result from recent nutritional deficiency which has been associated with availability of food and disease prevalence.

In comparison to the WHO standards, the sampled population HAZ distribution is platykurtic with lower and broader central peaks (see Figure 2). This is indicative of the population mean not being centered around the WHO recommended standards. Height for age *Z*-scores (HAZ) is an indicator for stunting represented by low HAZ and has been demonstrated to result from prolonged periods of either inadequate food intake, poor diet quality of morbidity from disease, or a combination of the same. Figure 2 shows distinct deviation from the WHO standard which may be indicative of either one or a combination of factors [2]. In this instance, boys have been shown to be more affected compared to girls.

Weight for age being an indicator of underweight is usually a composite of both WHZ and HAZ. This therefore also serves as an indicator of malnutrition which among the many causes chronic parasitism cannot be ruled out.

The study also showed that the number of boys affected by malnutrition was slightly higher compared to that of girls affected by malnutrition (see Table 6). In general, prevalence of malnutrition stood at 27.7% for wasting, 17.7% for underweight, and 6.94% for stunting with a majority of these cases occurring in the rural areas. This is a reflection of the 2008-2009 Kenya Demographic Health Survey for children under 5 years which showed that, nationwide, 35.3%, 6.7%, and 16.3% of the children were stunted, wasted, and underweight, respectively, and further suggested the greatest burden of malnutrition was in rural areas [2, 15].

The synergistic relationship between nutrition and infection can be attributed to the observed findings whereby either exposure to infections may be the cause of the malnutrition or the malnutrition predisposed the children making them more susceptible to infection. This is but a hypothetical deduction based on the study finding and thus further study is needed to ascertain the underlying cause of the observations made in this population

## 5. Conclusion

In conclusion, this study has demonstrated that the prevalence of STH and schistosomiasis in Mwea division in Kirinyaga County, Central Kenya is relatively low with a tendency to increase with age. While children in this age group were found to be infected with both* S. mansoni* and STH, prevalence was generally low (<6%), therefore not likely to have a major public health impact in this age group. Nevertheless, regular intervention will be necessary. A high prevalence of* Giardia lamblia* infections (15%), while interesting, was not surprising, as this infection is fairly common, in environments where hygiene is poor. This finding in particular suggests the* G. lamblia* is likely to be a major public health concern among children aged 5 years or less in Mwea, as they are at a high risk. It is, therefore, important to consider establishing an intervention program targeting this particular age group. The study further suggests the need for further investigations into other parasitic infections that cause ill health in this age group in the study area. While the prevalence of schistosomiasis and STH may have been low, these are likely to increase in prevalence given the conducive environment for transmission of these parasites in the area.

This study has also shown that hand washing practices, water purification methods, rural homes, and families with siblings above 5 years to are associated with infection in this age group. It is thus important to provide health education programmes for disease prevention, improved access to clean and safe water for domestic use, and appropriate sanitation.

Although the study was not able to establish a firm association between infection and malnutrition, the moderate prevalence of malnutrition in this age group cannot be ignored and the contribution of parasitic infections to the malnutrition cannot be entirely ruled out. It therefore calls for further investigations into the nutritional status of this age group to identify the underlying cause(s). Inclusion of nutrition in education is also recommended with a focus on families with preschool age children.

## Figures and Tables

**Table 1:** Summary of anthropometric descriptive statistics of the sampled study population.

	Mean	Confidence interval
*Age in months*		
Male (*n* = 183)	46.30 ± 10.01	(44.85–47.75) 95% CI
Female (*n* = 179)	46.93 ± 9.36	(45.55–48.30) 95% CI
Total 361	46.62 ± 9.68	(45.62–47.62) 95% CI
*Height in cm*		
Male (*n* = 183)	101.34 ± 6.43	(100.41–102.27) 95% CI
Female (*n* = 178)	102.23 ± 6.69	(101.24–103.21) 95% CI
Total: 361	101.78 ± 6.57	(101.10–102.45) 95% CI
*Weight in kg*		
Male (*n* = 183)	14.80 ± 2.06	(14.50–15.10) 95% CI
Female (*n* = 178)	14.61 ± 2.11	(14.30–14.92) 95% CI
Total: 361	14.71 ± 2.08	(14.49–14.92) 95% CI

*n* = total number of children.

**Table 2:** Age/sex distribution of the sampled study population (*n* = 361).

Age group	Female		Male		Total	
	Count	%	Count	%	Count	%
<2.5 years	14	3.88%	20	5.54%	34	9.42%
2.5–3.0 years	13	3.60%	16	4.43%	29	8.03%
3.0–3.5 years	24	6.65%	22	6.09%	46	12.74%
3.5–4.0 years	38	10.53%	39	10.80%	77	21.33%
4.0–4.5 years	44	12.19%	46	12.74%	90	24.93%
>4.5 years	45	12.47%	40	11.08%	85	23.55%
*Grand total*	*178*	*49.31%*	*183*	*50.69%*	*361*	*100.00%*

**Table 3:** Frequency distribution of socioeconomic characteristics of the sampled study population.

Attribute	Response	Frequency	% frequency
Knowledge of disease transmission	No	114	31.6%
	Yes	247	68.4%

Geophagy (soil eating)	No	74	20.5%
	Yes	287	79.5%

Hand washing (child)	Never	118	32.7%
	Sometimes	213	59.0%
	Always	30	8.3%

Shoe wearing	Sometimes	325	90.0%
	Always	36	10.0%

Water source (domestic)	River/canal	292	80.9%
	Borehole	43	11.9%
	Piped	26	7.2%

River bathing child	No	98	27.1%
	Yes	263	72.9%

Water purification method	None	71	19.7%
	Filtration	115	31.9%
	Boiling	79	21.9%
	Chlorination	96	26.6%

Bathroom waste water disposal	Open ground	275	76.2%
	Latrine	86	23.8%

Employment status (father)	No	75	20.8%
	Yes	286	79.2%

Employment status (mother)	No	236	65.4%
	Yes	125	34.6%

Home ownership	Self-own	208	57.6%
	Rental	153	42.4%

Home location classification	Rural	284	78.7%
	Urban	77	21.3%

Family with children above 5 yrs	No	249	69.0%
	Yes	112	31.0%

House type	Rural	289	80.1%
	Wooden	8	2.2%
	Iron sheets	12	3.3%
	Brick/stone	52	14.4%

**Table 4:** Prevalence of parasitic infections in sampled study population in Mwea Division.

Row labels	Frequency	Percentage	Boys	Percentage	Girls	Percentage
*Ancylostoma duodenale*	2	0.55%	1	0.53%	1	0.58%
*Ascaris lumbricoides*	12	3.05%	6	3.19%	5	2.89%
*E. coli*	7 (7)^*∗*^	3.88%	5	2.66%	9	5.20%
*E. histolytica*	1	0.28%	0	0.00%	1	0.58%
*E. vermicularis*	3	0.83%	1	0.53%	2	1.16%
*G. lamblia*	54	14.68%	29	15.43%	25	13.87%
*H. nana*	9 (4)^*∗*^	3.60%	4	2.13%	9	5.20%
*No infection*	253	66.48%	123	65.43%	117	67.63%
*Schistosoma mansoni*	18 (2)^*∗*^	5.54%	17	9.04%	3	1.73%
*Trichuris trichiura*	2 (2)^*∗*^	1.11%	2	1.06%	2	1.16%
*Grand total*	*361*	*100.00%*	*188*	*100.00%*	*173*	*100.00%*

^*∗*^Occurrence as multiple infections.

**Table 5:** Frequency distribution of parasitic infections per age groups.

Age Group	*S. mansoni*	Hookworm	*A. lumbricoides*	*T. trichiura*	*G. lamblia*	*H. nana*	*E.vermicularis*	*E. histolytica*	*E. coli*
<2.5 yrs	1	0	1	0	4	1	0	0	3
2.5–3 yrs	1	0	1	0	1	0	0	0	1
3.0–3.5 yrs	2	0	1	0	6	0	1	0	3
3.5–4.0 yrs	3	1	1	0	9	1	0	1	5
4.0–4.5 yrs	5	1	3	4	15	7	2	0	0
4.5–5 yrs	8	0	4	0	19	4	0	0	2
*Grand total*	*20*	*2*	*11*	*4*	*54*	*13*	*3*	*1*	*14*

**Table 6:** Prevalence of malnutrition in PSAC in Mwea Division based on the children's *Z*-scores.

	Mean *Z*-score values	95% confidence interval	% of moderately malnourished children	% of severely malnourished children
*WAZ*				
Male (*n* = 183)	−0.66 ± 1.08	(−0.82–−0.51) 95% CI	14.2% underweight (<−2*z*)	2.2% severe underweight (<−3*z*)
Female (*n* = 178)	−0.64 ± 1.07	(−0.79–−0.48) 95% CI	11.8% underweight (<−2*z*)	1.1% severe underweight (<−3*z*)

*HAZ*				
Male (*n* = 183)	−0.11 ± 1.37	(−0.31–0.09) 95% CI	8.2% stunted (<−2*z*)	0.5% severe stunted (<−3*z*)
Female (*n* = 178)	0.15 ± 1.25	(−0.04–0.33) 95% CI	3.4% stunted (<−2*z*)	0.16% severe stunted (<−3*z*)

*WHZ*				
Male (*n* = 183)	−0.90 ± 1.12	(−1.07–−0.74) 95% CI	20.8% wasted (<−2*z*) 0.0% obese (>2*z*)	3.8% severe wasted (<−3*z*)
Female (*n* = 178)	−1.10 ± 1.04	(−1.25–−0.95) 95% CI	20.2% wasted (<−2*z*) 0.0% obese (>2*z*)	3.4% severe wasted (<−3*z*)

CI = confidence interval, *n* = total number of children, and *z* = *Z*-score.

**Table 7:** Factors associated with the general prevalence of infection in preschool age children in Mwea division: a binary logistic regression model.

Variable	OR	(*P* value)	95% CI	
Knowledge of disease transmission	.862	.635	.629	2.137
Geophagy	.975	.947	.459	2.072
Hand washing				
Never	6.478	.010^*∗*^	1.553	27.015
Sometimes	3.401	.093	.817	14.167
Shoe wearing	.405	.155	.117	1.406
Water source				
Borehole	.621	.566	.122	3.167
River/canal	.194	.088	.029	1.278
Water purification method				
None	3.602	.008^*∗*^	1.397	9.288
Filtration	.778	.537	.351	1.725
Boiling	1.272	.572	.552	2.932
Family with children above 5 years	.390	.007^*∗*^	1.293	5.088
Constant	6.206	.216		

OR = odds ratio, CI = confidence interval, and *∗* = variables with statistical significance.

**Table 8:** Prevalence of malnutrition by age groups in PSAC in Mwea Division.

Age (months)	Total number	Severe wasting (<−3 *z*-score)		Moderate wasting (≥−3 and <−2 *z*-score)		Severe underweight (<−3 *z*-score)		Underweight (≥−3 and <−2 *z*-score)		Severe stunted (<−3 *z*-score)		Moderate stunted (≥−3 and <−2 *z*-score)	
		Number	%	Number	%	Number	%	Number	%	Number	%	Number	%
6–17													
18–29	34	1	2.9	5	14.7	0	0.0	0	0.0	0	0.0	0	0.0
30–41	71	3	4.2	8	11.3	1	1.4	4	4.2	1	1.4	2	2.8
42–53	163	4	2.5	31	19.0	6	2.5	40	20.2	1	0.6	14	8.6
54–59	93	5	5.4	17	18.3	1	1.1	12	11.8	0	0.0	6	5.4

Total	361	13	3.6	61	16.9	8	2.2	56	15.5	2	0.8	22	15.5

## Abbreviations

ANOVA: Analysis of variance
CBRD: Centre for Biotechnology and Research Development
CDC: Centre for Disease Control
CI: Confidence interval
HAZ: Height for Age *Z*-scores
ITROMID: Institute of Tropical Medicine and Infectious Diseases
JKUAT: Jomo Kenyatta University of Agriculture and Technology
KEMRI: Kenya Medical Research Institute
MDA: Mass drug administration
NACOSTI: National Commission of Science, Technology and Innovation
NTDs: Neglected Tropical Diseases
PSAC: Preschool age children
SERU: Scientific and Ethics Review Unit
SPSS: Statistical Package for Social Sciences
STH: Soil-transmitted helminthes (~iases)
SSA: Sub-Saharan Africa
WAZ: Weight for age *Z*-scores
WHO: World Health Organization
WHZ: Weight for height *Z*-scores.
